# ZnO@TiO_2_/PDMS Superhydrophobic Antibacterial Coating with Photocatalytic Activity, Durability, and Self-Cleaning Properties

**DOI:** 10.3390/ma19112380

**Published:** 2026-06-03

**Authors:** Shuyu Yuan, Yuan Feng, Shuaichao Liang, Huidong Cai, Qingge Feng

**Affiliations:** 1 Guangxi Universities Key Laboratory of Environmental Protection, School of Resources, Environment and Materials, Guangxi University, Nanning 530004, China; yuanshuyu_1@163.com (S.Y.); fengy_cup@163.com (Y.F.); m17877988861@163.com (S.L.); 2 School of Environmental Science and Engineering, Guangdong University of Petrochemical Technology, Maoming 525000, China

**Keywords:** superhydrophobicity, photocatalysis, self-cleaning, antibacterial adhesion, antibacterial

## Abstract

**Highlights:**

- The ZT/PDMS superhydrophobic coating was successfully fabricated by a facile method and exhibited a water contact angle of 153.5°.
- ZT/PDMS exhibits outstanding durability, chemical stability and self-cleaning properties.
- Synergistic effects stem from “anti-adhesion, sterilization, and self-cleaning”.

**Abstract:**

Superhydrophobic antibacterial coatings offer an effective approach to overcoming the limitations of single anti-adhesion or bactericidal strategies; however, it remains a great challenge to develop such coatings with long-term durability and high bactericidal performance. In this study, a ZT/PDMS composite coating was successfully fabricated by directly mixing ZnO@TiO_2_ with PDMS. Benefiting from the low surface energy of polydimethylsiloxane (PDMS) and the coral-like micro/nanostructured rough morphology generated by the incorporation of ZnO@TiO_2_ nanoparticles, the coating exhibited excellent superhydrophobic properties, with a water contact angle of 153.5°. The proposed fabrication method showed good adaptability to various substrates, and the resulting coating demonstrated outstanding durability and self-cleaning performance. Notably, the coating retained superhydrophobicity after six abrasion cycles, and the water contact angle remained above 140° after immersion in solutions with pH ranging from 1 to 13 for 7 days. The ZT/PDMS composite coating achieved an antibacterial adhesion rate of 87.98% and 80.11% against *Acinetobacter baumannii* (*A. baumannii*) and *Staphylococcus aureus* (*S. aureus*), respectively. Under UV and visible light irradiation, its bactericidal efficiency exceeded 90%. The excellent antibacterial performance of the coating was attributed to the synergistic effects of anti-adhesion, active sterilization (Zn^2+^ release and ROS generation), and self-cleaning. This study provides a facile and effective strategy for the development of efficient and durable multifunctional antibacterial coatings.

## 1. Introduction

Bacterial contamination has become an increasingly critical issue in healthcare [1], food processing [2], and marine engineering [3], posing substantial threats to human health and socioeconomic development. Bacteria can spread through the air, water, and the surfaces of objects, leading to infectious diseases and cross-contamination. Moreover, bacterial adhesion and proliferation on material surfaces can result in the formation of biofilms, which exhibit high resistance to conventional sterilization methods [4]. Traditional antibacterial strategies based on chemical disinfectants and antibiotics are associated with environmental concerns and the growing risk of antimicrobial resistance, limiting their effectiveness for long-term and sustainable antibacterial protection [5,6,7]. Consequently, antibacterial materials have attracted considerable research interest owing to their prolonged efficacy and lower likelihood of inducing bacterial resistance [8,9,10,11].

Superhydrophobic anti-adhesion strategies, generally characterized by a water contact angle greater than 150° and a sliding angle below 10°, rely on the synergistic combination of micro/nanostructured surface roughness and low surface energy [12,13]. According to the Cassie–Baxter wetting model, such surfaces trap an air layer at the solid–liquid interface, significantly reducing bacterial contact and inhibiting initial adhesion and biofilm formation, while also endowing the material with self-cleaning properties [14].

Bactericidal strategies, particularly those based on metal oxides such as CuO, ZnO, and TiO_2_, have gained widespread attention because of their excellent stability, long-term antibacterial activity, and low propensity to induce resistance [15,16]. Their antibacterial mechanisms primarily involve two pathways: (i) the release of metal ions, which adsorb onto negatively charged bacterial membranes, disrupt membrane integrity and transmembrane potential, and interfere with intracellular biomolecules such as proteins and DNA [17,18]; and (ii) the photocatalytic generation of reactive oxygen species (ROS) under light irradiation, leading to oxidative damage of bacterial membranes, proteins, and nucleic acids [19,20,21].

Despite their advantages, both anti-adhesion and bactericidal strategies exhibit inherent limitations when applied independently. Anti-adhesion surfaces primarily function by preventing bacterial attachment but lack active bactericidal capability, making them vulnerable once the surface barrier is compromised [22]. In contrast, bactericidal surfaces may accumulate dead bacterial residues, which can reduce long-term antibacterial efficiency and potentially induce inflammatory responses [23,24,25]. Therefore, the development of multifunctional antibacterial systems that integrate anti-adhesion and bactericidal mechanisms is of significant importance. Superhydrophobic antibacterial coatings that combine these functionalities with self-cleaning capability and long-term durability represent a promising approach for practical applications [26,27]. Zhang et al. constructed a superhydrophobic antibacterial coating with hierarchical micro/nanostructured roughness on cotton fabric via a facile one-step dip-coating method using chitosan (CS), stearic acid (SA), and Cu-MOFs/Zn-MOFs as precursor materials [28]. Benefiting from the synergistic effect of the low surface energy modification and the rough micro/nanostructure generated by MOF particles, the coating exhibited excellent superhydrophobicity, with a water contact angle exceeding 165°, along with outstanding self-cleaning capability that effectively inhibited bacterial adhesion. Meanwhile, chitosan and the sustained release of Cu^2+^ and Zn^2+^ ions worked synergistically to provide strong bactericidal activity, achieving antibacterial efficiencies of 94.8% against *E. coli* and 97.5% against *S. aureus*. In addition, the coating demonstrated favorable mechanical durability and chemical corrosion resistance, indicating its potential for long-term antibacterial applications.

Superhydrophobic coatings are typically fabricated by first modifying nanoparticles with hydrophobic agents and then incorporating them into a binder matrix. Fluorinated compounds are conventionally employed to impart low surface energy. For example, Ren et al. modified SiO_2_ with 1H,1H,2H,2H-perfluorooctyltriethoxysilane, mixed the resulting modified nanoparticles with an acid-catalyzed silica sol–gel precursor, then sprayed the mixture once onto a glass substrate, and dried it at room temperature to obtain a superhydrophobic coating [29]. Liu et al. integrated the silica nanoparticles modified by perfluorodecyl triethoxysilane (FAS-17) with fluorinated resin and polymethyl methacrylate (PMMA) to prepare a superhydrophobic coating [30]. Although these approaches yield coatings with pronounced hydrophobicity, they are often associated with complex processing, high cost, and potential toxicity of fluorinated substances, which raise additional concerns due to their environmental persistence and bioaccumulation risks [31,32].

Polydimethylsiloxane (PDMS), a fluorine-free silicone polymer, contains abundant Si-O-Si backbones and hydrophobic methyl (-CH_3_) groups, which confer intrinsically low surface free energy. In addition, PDMS exhibits excellent flexibility, chemical stability, resistance to photodegradation, and biocompatibility. It also functions as an effective binder, enabling stable anchoring of functional nanoparticles onto various substrates and thereby enhancing the mechanical robustness of the resulting coatings [33,34].

To address the limitations of single antibacterial strategies and to provide a simple preparation method, a ZnO@TiO_2_/PDMS superhydrophobic composite coating was successfully fabricated in this work by directly mixing ZnO@TiO_2_ with PDMS. The coating exhibited superhydrophobic performance, excellent mechanical and chemical stability, outstanding self-cleaning performance, high antibacterial adhesion efficiency, and over 90% bactericidal activity. Furthermore, the underlying synergistic mechanism of “anti-adhesion, active sterilization, and self-cleaning” was elucidated. This study provides a promising strategy for developing long-lasting, high-performance antibacterial coatings.

## 2. Materials and Methods

### 2.1. Materials

Zinc oxide (ZnO) nanoparticles and tetrabutyl titanate (Ti(OC_4_H_9_)_4_, 98.0%) were purchased from Shanghai Macklin Biochemical Technology Co., Ltd., Shanghai, China. Ammonium hydroxide (NH_3_·H_2_O, 25%~28%) and n-hexane were obtained from Guangdong Guanghua Technology Co., Ltd., Shantou, China. Ethanol (EtOH, 99%) was purchased from Chengdu Kelong Chemical Co., Ltd., Chengdu, China. Polydimethylsiloxane (PDMS) was supplied by Dow Corning Corporation, Midland, MI, USA. *Acinetobacter baumannii* (*A. baumannii*) was purchased from Guangdong Microbial Culture Center, Guangzhou, China.

### 2.2. Synthesis of ZnO@TiO_2_ Nanoparticles

The ZnO@TiO_2_ core–shell nanoparticles (ZT) were prepared via a sol–gel method [35]. A total of 1 g of ZnO nanoparticles was added to a mixture of 250 mL of EtOH and 3 mL of NH_3_·H_2_O and ultrasonicated for 30 min to achieve uniform dispersion. A solution of 2.5 mL of TBOT was slowly added to the above dispersion, and the reaction mixture was continuously stirred for 48 h. The resulting product was washed with EtOH, dried at 60 °C for 24 h, and finally calcined at 500 °C for 2 h.

### 2.3. Preparation of ZT/PDMS Composite Coating

Substrate pretreatment: Glass slides were placed in a beaker containing EtOH and ultrasonicated for 2 h to remove surface impurities. The cleaned glass slides were then dried for later use.

**Figure 1:**
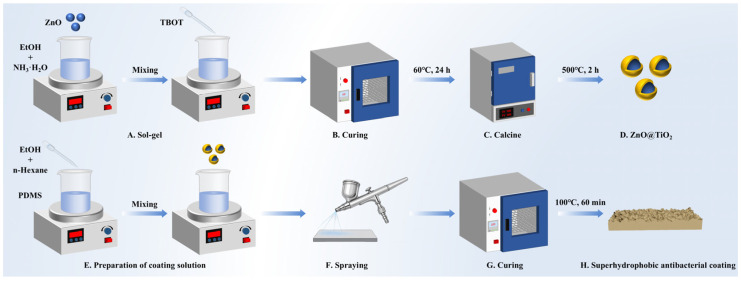
Schematic illustration of the fabrication of the ZT/PDMS composite coating.

Coating preparation: As shown in Figure 1, the PDMS prepolymer and curing agent were mixed at a mass ratio of 10:1, stirred for 30 min to homogenize, and then degassed under vacuum for 30 min to remove bubbles. The PDMS mixture was subsequently blended with EtOH and n-hexane at a mass ratio of 1:5:5. To determine the optimal ratio of ZnO@TiO_2_ to PDMS, coatings with different mass ratios (ZT:PDMS = 0.25:1, 0.5:1, 0.75:1, 1:1, and 1.25:1) were prepared and their water contact angles were measured. As shown in Figure S2, the 1:1 ratio exhibited the highest water contact angle (155.2°), indicating the best hydrophobic performance. Accordingly, ZT nanoparticles were then added at a mass ration of m (ZT:PDMS) = 1:1, and the mixture was stirred and ultrasonicated until homogeneous. Finally, the resulting suspension was sprayed onto the pretreated glass slides using a spray gun and cured at 100 °C for 60 min to obtain the ZnO@TiO_2_/PDMS (ZT/PDMS) composite coating. The distance between the nozzle and the substrate was set at 20 cm, and the spraying duration was 10 s.

### 2.4. Characterization

The crystal structure of the synthesized materials was analyzed by X-ray diffraction (XRD) using the Bruker D8 Advance diffractometer (Bruker, Bremen, Germany) with Cu Kα radiation. Fourier-transform infrared (FTIR) spectra were measured on a Shimadzu IRTracer-100 spectrometer (Shimadzu, Kyoto, Japan) over the wavenumber range of 400–4000 cm^−1^ to identify the chemical bonds and functional groups. X-ray photoelectron spectroscopy (XPS) measurements were performed on a Thermo Scientific K-Alpha spectrometer (Thermo Scientific, Waltham, MA, USA) equipped with monochromatic Al Kα radiation (1486.6 eV). The binding energy scale was calibrated by setting the adventitious C 1s peak to 284.8 eV. The surface morphology and microstructure of the coatings were examined by scanning electron microscope (SEM, Carl Zeiss, Oberkochen, Germany) coupled with an energy dispersion spectrometer (EDS) for elemental analysis. Atomic force microscopy (AFM) images were obtained with a Hitachi 5100N instrument (Hitachi, Tokyo, Japan) to evaluate the surface topography and roughness. The concentration of Zn^2+^ ions released from the coatings was determined by inductively coupled plasma mass spectrometer (ICP-MS, ICAP 7000, Thermo Scientific, Waltham, MA, USA). Electron paramagnetic resonance (EPR) spectra were recorded on a Bruker EMXplus-6/1 spectrometer (Bruker, Bremen, Germany) to detect reactive oxygen species (ROS). Water contact angles (WCAs) and sliding angles (SAs) were measured using a DSA100 drop shape analyzer (Krüss, Hamburg, Germany) to assess the wetting behavior of the coatings.

### 2.5. Mechanical Stability

The ZT/PDMS composite coating was placed face down on a piece of 600-grit sandpaper, and a 100 g weight was loaded onto the back of the coating. The coating was then pulled vertically at a constant speed over a distance of 20 cm per cycle. The WCA and SA of the coating were measured to monitor their changes with increasing abrasion cycles, thereby evaluating the mechanical stability of the coating [36].

### 2.6. Chemical Stability

A series of solutions with pH of 1, 3, 5, 7, 9, 11, and 13 was prepared using HNO_3_ solution and NH_3_·H_2_O. The ZT/PDMS composite coating was immersed in each of these pH solutions and allowed to stand for 7 days. The chemical stability of the coating was evaluated by measuring the changes in its WCA and SA after exposure to solutions of different pH.

### 2.7. Self-Cleaning Performance

The prepared ZT/PDMS composite coating was placed at a tilt angle of 20°. Aluminum ash was used as a model contaminant and was uniformly distributed on the coating surface. A water droplet was released from a height of 10 cm above the coating surface, and the removal of aluminum ash by the rolling droplet was observed and recorded photographically. A pristine glass slide was used as a reference for comparison to more clearly demonstrate the self-cleaning performance of the coating.

Dye solutions of methyl orange (MO), rhodamine B (RhB), and methylene blue (MB) were prepared. The ZT/PDMS composite coating and a pristine glass slide were immersed in each dye solution for three cycles. The residual dye solution on the coating surface was observed and photographed to evaluate the self-cleaning performance of the coating.

### 2.8. Photocatalytic Experiment

A 500 W xenon lamp was used as the visible light source, and MO was selected as the model pollutant. The coating was immersed in 100 mL of MO aqueous solution (20 mg/L). The mixture was first stirred magnetically in the dark for 30 min to reach adsorption–desorption equilibrium. Subsequently, the solution was irradiated under visible light for 60 min. At 10 min intervals, aliquots were sampled, filtered through a 0.45 μm nylon syringe filter, and the absorbance was measured at 464 nm using a UV-Vis spectrophotometer to determine the solution concentration. The degradation rate (η) of MO was calculated according to the following equation:

(1) $$\eta \;=\;\frac{C_{0}\;-{\;C}_{t}}{C_{0}}\;\times \;100\%$$

where C_t_ is the concentration of MO at t min, C_0_ is the initial concentration of MO.

### 2.9. Zn^2+^ Release

To evaluate the Zn^2+^ release performance of the coating, the ZT/PDMS composite coating was placed in 50 mL of PBS and incubated at 37 °C under dark conditions. At predetermined time intervals, 3 mL of the solution was withdrawn, filtered through a 0.45 μm nylon syringe filter, diluted with 3% nitric acid, and then analyzed for Zn^2+^ concentration by ICP-MS.

### 2.10. Antibacterial Adhesion Performance

In this study, *A. baumannii* and *S. aureus* were selected as the model bacteria for antibacterial evaluation. *A. baumannii* is a typical Gram-negative pathogen widely distributed in healthcare settings. It is highly virulent, causing severe infections such as pneumonia, respiratory and urinary tract infections, and wound infections [37]. Alarmingly, approximately 45% of clinical isolates of *A. baumannii* worldwide are multidrug-resistant. Given its critical threat to public health, the WHO has placed *A. baumannii* at the top of its priority pathogen list [38,39]. Therefore, selecting *A. baumannii* helps assess the practical application potential of the developed antibacterial material against highly pathogenic and multidrug-resistant bacteria. In contrast, *S. aureus*, is a representative Gram-positive pathogen.

All experimental materials and reagents were sterilized in an autoclave at 120 °C for 20 min, while the coating samples were disinfected by UV irradiation before the tests. Coating samples (1 cm × 1 cm) were placed in a 24-well plate, and 1 mL of *A. baumannii* suspension at a concentration of 10^7^ CFU/mL (*S. aureus* suspension at a concentration of 10^8^ CFU/mL) was added to each well. The samples were co-incubated with the bacterial suspension at 37 °C in the dark for 6 h. After incubation, the samples were gently rinsed with sterile PBS to remove unadherent bacteria. The rinsed coatings were then transferred to centrifuge tubes containing 1 mL of sterile PBS and sonicated for 5 min to fully detach the bacteria adhered to the coating surfaces. Finally, 100 µL of the serially diluted bacterial suspension was spread onto LB agar plates and incubated at 37 °C for 18–24 h, and the number of colony-forming units (CFUs) on each plate was recorded. A pristine glass slide was used as a control and subjected to the same procedure. All experiments were performed in triplicate to ensure the reliability of the results. The antibacterial adhesion rate of the coating was calculated by the following formula:

(2) $$\mathrm{Antibacterial}\;\mathrm{adhesion}\;\mathrm{rate}\;(\%)\;=\;\frac{C_{\mathrm{control}\;}-\;C}{C_{\mathrm{control}}}\;\times \;100\%$$

where C and C_control_ refer to the bacterial counts in the experimental and control samples, respectively.

### 2.11. Antibacterial Performance

The pretreatment of materials, reagents, and samples prior to the antibacterial performance test followed the procedure described in Section 2.8. Coating samples (1 cm × 1 cm) were placed in a 24-well plate, and 1 mL of *A. baumannii* suspension at a concentration of 10^6^ CFU/mL (*S. aureus* suspension at a concentration of 10^7^ CFU/mL) was added to each well. The samples were co-incubated with the bacterial suspension at 37 °C for 2 h under three different conditions: darkness, UV light (30 W), and visible light (60 W). After incubation, the bacterial suspension in each well was sampled and spread onto LB agar plates, followed by incubation at 37 °C for 18–24 h. All experiments were performed in triplicate to ensure the reliability of the results.

## 3. Results and Discussion

### 3.1. Materials Characterization

The TEM, XRD, and FTIR results of the ZnO@TiO_2_ nanoparticles are presented in Figure S1. Figure S1a reveals a clear core–shell structure, with the shell exhibiting a lattice fringe spacing of 0.35 nm, corresponding to the (101) plane of TiO_2_. As shown in Figure S1b, the diffraction peaks observed at 2θ = 31.77°, 34.42° and 62.86° corresponded to the (100), (002) and (103) crystal planes of hexagonal crystalline ZnO (PDF#73-1764) [40]. No diffraction peaks of TiO_2_ were detected, which could be ascribed to its low content resulting in weak peak intensities. In the FTIR spectrum (Figure S1c), the absorption band of zinc oxide at 441 cm^−1^ was attributed to the stretching vibration of the Zn-O bond. Upon the incorporation of TiO_2_, this band shifted toward a higher wavenumber. Collectively, these observations confirm the formation of the ZnO@TiO_2_ nanoparticles.

**Figure 2:**
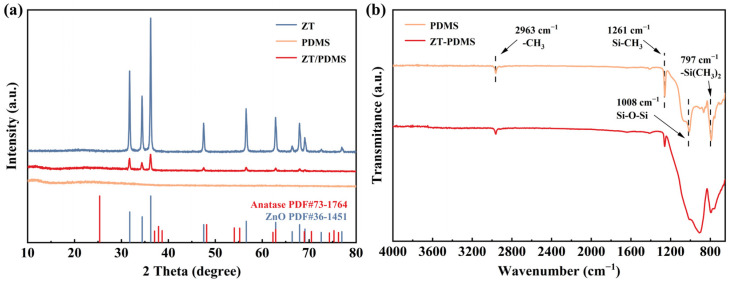
(**a**) XRD patterns and (**b**) FTIR spectra of ZT, PDMS coating, and ZT/PDMS composite coating.

XRD and FTIR analyses were conducted to characterize the chemical compositions of pure PDMS and the ZT/PDMS composite coating, and the results are presented in Figure 2. As shown in Figure 2a, the ZT/PDMS composite coating displayed the same diffraction peaks as the ZnO@TiO_2_ nanoparticles, and no additional peaks were observed. This indicated that the introduction of PDMS did not alter the crystal structure of the ZT nanoparticles. Notably, the intensity of the ZnO diffraction peaks in the ZT/PDMS composite coating was lower than that of the pristine ZT nanoparticles, which can be attributed to the coverage of the amorphous PDMS.

The FTIR spectra in the wavenumber range of 650–4000 cm^−1^ are shown in Figure 2b. For the pure PDMS coating, the absorption bands at 2963 cm^−1^ and 1261 cm^−1^ corresponded to the stretching vibration of -CH_3_ [41], the band at 1008 cm^−1^ was assigned to the asymmetric stretching vibration of Si-O-Si bonds [42], and the band at 797 cm^−1^ arose from the vibration mode of -Si(CH_3_)_2_ [43]. The FTIR spectrum of the ZT/PDMS composite coating exhibited the same characteristic absorption peaks, indicating that no significant chemical reaction occurred between ZT and PDMS.

**Figure 3:**
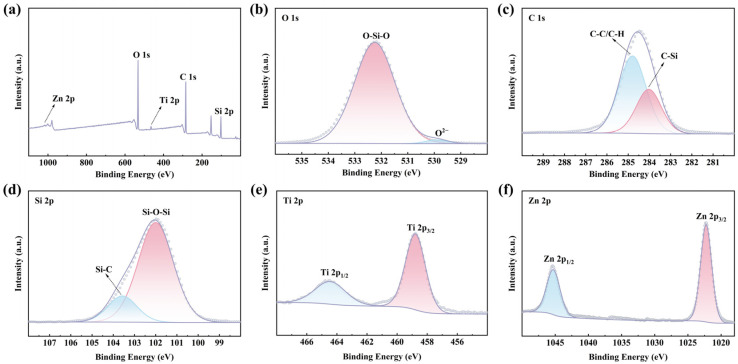
XPS spectra of ZT/PDMS composite coating: (**a**) survey spectrum; (**b**) high-resolution O 1s spectrum; (**c**) high-resolution C 1s spectrum; (**d**) high-resolution Si 2p spectrum; (**e**) high-resolution Ti 2p spectrum; (**f**) high-resolution Zn 2p spectrum.

As shown in Figure 3a, the survey spectrum of the ZT/PDMS composite coating exhibited characteristic peaks corresponding to Zn 2p, O 1s, Ti 2p, C 1s, and Si 2p, indicating the presence of Zn, O, Ti, C, and Si elements. The detailed surface atomic concentration fractions (%) obtained from XPS analysis are listed in Table S1. In the O 1s spectrum (Figure 3b), the peaks at 532.25 eV and 529.9 eV were assigned to the O-Si-O bond and lattice oxygen, respectively [44,45]. The C 1s spectrum (Figure 3c) showed peaks at 284.8 eV and 284.03 eV, corresponding to the C-C/C-H and C-Si bonds, respectively [33,46]. In the Si 2p spectrum (Figure 3d), the peaks at 103.57 eV and 102.00 eV were attributed to the Si-C and Si-O-Si bonds, respectively [47]. In the Ti 2p spectrum (Figure 3e), the peaks at 458.20 eV and 463.91 eV were assigned to Ti 2p_3/2_ and Ti 2p_1/2_, respectively [48]. The Zn 2p spectrum (Figure 3f) exhibited peaks at 1021.2 eV and 1044.13 eV, which corresponded to Zn 2p_3/2_ and Zn 2p_1/2_, respectively [49]. These results collectively confirmed the successful integration of ZT and PDMS.

**Figure 4:**
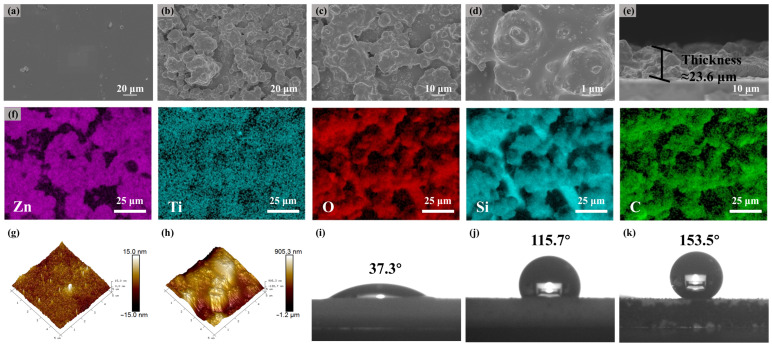
SEM images of (**a**) the PDMS coating and (**b**–**d**) the ZT/PDMS composite coating; (**e**) cross-sectional SEM images of the ZT/PDMS composite coating; (**f**) EDS mapping image of the ZT/PDMS composite coating; AFM images of (**g**) the PDMS coating and (**h**) the ZT/PDMS composite coating; Water contact angles of (**i**) a glass slide, (**j**) the PDMS coating, and (**k**) the ZT/PDMS composite coating.

The surface microstructure of the coatings was characterized by scanning electron microscopy (SEM). As shown in Figure 4a, the PDMS coating exhibited a relatively smooth and uniform surface without noticeable protrusions or hierarchical features. In contrast, the ZT/PDMS composite coating displayed a typical coral-like micro/nanostructured rough morphology (Figure 4b–d). This hierarchical roughness, generated by the aggregation of nanoparticles, can effectively trap air within the surface asperities, forming a stable air cushion that significantly reduces the actual liquid–solid contact area [50].

Cross-sectional SEM images (Figure 4e) further revealed that the ZT/PDMS composite coating adhered tightly to the substrate and exhibited a uniform thickness of approximately 23.6 μm. The element distribution of the ZT/PDMS coating was further investigated by energy-dispersive spectroscopy (EDS) elemental mapping. As shown in Figure 4f, EDS analysis demonstrated the homogeneous distribution of Zn, Ti, O, Si, and C elements across the coating surface, indicating the uniform dispersion of ZT nanoparticles within the PDMS matrix.

Atomic force microscopy (AFM) measurements were performed to quantitatively evaluate the surface morphology and roughness of the coatings, and the results are presented in Figure 4g,h. The PDMS coating exhibited an average surface roughness of only 1.88 nm, confirming its smooth surface characteristics. After incorporation of ZT nanoparticles, the average roughness of the ZT/PDMS composite coating increased significantly to 175 nm. The AFM findings were consistent with the SEM observations, collectively confirming that the introduction of ZT nanoparticles effectively constructed a hierarchical micro/nanostructured rough surface on the coating, thereby increasing the surface roughness.

Figure 4i–k presented the wettability of the pristine glass substrate and the prepared coatings. The untreated glass substrate exhibited a hydrophilic surface with a WCA of approximately 37.3°. In contrast, the PDMS coating showed a WCA of 115.7° due to the intrinsically low surface energy of PDMS, indicating a transition from hydrophilicity to hydrophobicity. For the ZT/PDMS composite coating, the incorporation of ZT nanoparticles created a coral-like micro/nanostructured rough surface, which substantially enhanced the surface roughness. As a result, the WCA further increased to 153.5°, demonstrating the formation of a superhydrophobic surface. The ZT/PDMS coating exhibited excellent superhydrophobic properties owing to the synergistic effect of low surface energy contributed by PDMS (attributed to its abundant -Si-O-Si- backbones and hydrophobic -CH_3_ groups) and enhanced surface roughness provided by the micro/nano structures formed by ZT nanoparticles [33,34].

### 3.2. Durability and Stability of ZT/PDMS Composite Coating

**Figure 5:**
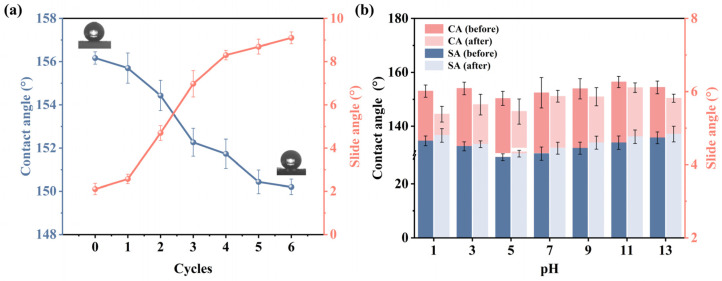
WCA and SA of ZT/PDMS coating (**a**) with increasing wear cycles and (**b**) after 7 d of immersion in various pH solutions.

Figure 5a illustrates the variation in WCA and SA of the ZT/PDMS composite coating as a function of abrasion cycles. As the number of abrasion cycles increased, the contact angle showed a gradual decrease. This decline was primarily attributed to the partial destruction of the surface micro/nanostructured roughness during abrasion, resulting in reduced surface roughness [50]. In addition, wear of the PDMS matrix exposed some hydrophilic nanoparticles to the coating surface, thereby increasing the local surface energy and further weakening the hydrophobicity [51]. After five abrasion cycles, the contact angle of the coating decreased from 156.2° to 150.7°, while still remaining superhydrophobic. Corresponding to the decrease in contact angle, the sliding angle exhibited a gradual increasing trend with increasing abrasion cycles. The initial sliding angle was approximately 2°, indicating excellent droplet mobility on the coating surface. As abrasion progressed, the sliding angle continuously increased, reaching 8.7° after 5 abrasion cycles.

Overall, although the ZT/PDMS composite coating exhibited some loss of hydrophobicity during abrasion, it retained superhydrophobic characteristics after approximately 5 abrasion cycles, demonstrating favorable mechanical durability. In addition, adhesion tests performed on the ZT/PDMS composite coating according to ASTM regulations revealed an acceptable 4B adhesion rating, as illustrated in Figure S3. The interaction between ZnO@TiO_2_ and PDMS involves both physical electrostatic forces and chemical bonding, which enables the ZT nanoparticles to be tightly anchored onto the substrate, thereby contributing to the mechanical stability of the coating [52]. This mechanical stability could be further attributed to the flexibility of the Si-O-Si backbone within the PDMS matrix, which dissipates mechanical stress through elastic deformation and thereby mitigates damage to the micro/nanostructures. Furthermore, the strong adhesive nature of PDMS enhances the interfacial bonding strength between the coating and the substrate, effectively preventing coating delamination during friction, thus improving the mechanical stability of the coating [53].

Figure 5b presents the variations in WCA and SA of the ZT/PDMS composite coating before and after immersion in solutions with different pH for 7 days. After immersion in various pH solutions, the WCA of the coating decreased slightly overall but remained above 140°, indicating that its hydrophobic properties were essentially maintained. The deterioration was attributed to the chemical reaction of PDMS with alkalis and the acid-induced corrosion of the ZT nanoparticles [54]. Meanwhile, the SA exhibited only a small variation, remaining at a low level of approximately 5°, which further confirmed that the coating retained good hydrophobicity after exposure to solutions of different pH.

The excellent chemical stability of the ZT/PDMS composite coating could be attributed to two main factors. First, the micro/nanostructured rough surface could trap a stable air cushion at the solid–liquid interface, which effectively inhibited the penetration of acidic or alkaline solutions into the coating interior [55]. Second, PDMS possessed inherent chemical stability, which provided additional protection for the embedded nanoparticles against chemical attack [56].

A comparison of the WCA changes under different pH conditions revealed that the coating exhibited superior stability in alkaline environments. Under both strongly acidic (pH = 1) and strongly alkaline (pH = 13) conditions, only slight variations in contact angle were observed. In acidic solutions (pH = 1–5), the contact angle decreased by approximately 2.4–6.3°, and under strongly acidic conditions (pH = 1), the contact angle dropped below 150°, indicating a partial loss of superhydrophobicity. In contrast, under alkaline conditions (pH = 9–13), the contact angle decreased by only 0.5–2.2°, while the coating maintained excellent superhydrophobic behavior. These results suggest that the ZT/PDMS composite coating possesses stronger resistance to alkaline environments. In summary, the ZT/PDMS composite coating maintained high contact angles and low sliding angles after immersion in solutions ranging from pH 1 to 13, demonstrating the excellent ability to maintain stable hydrophobic performance under harsh acidic and alkaline conditions.

### 3.3. Adaptability and Self-Cleaning Performance of ZT/PDMS Composite Coating

**Figure 6:**
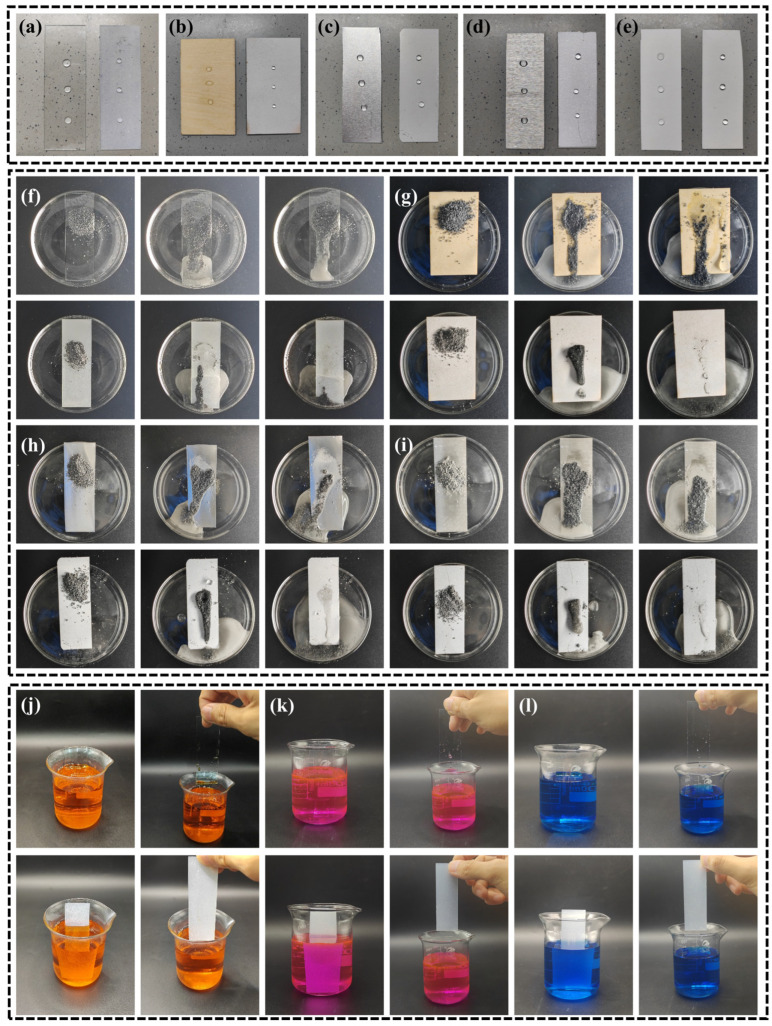
Hydrophobicity of coatings prepared on different substrates: (**a**) glass slide, (**b**) wood, (**c**) aluminum, (**d**) zinc, and (**e**) paper; Self-cleaning performance of coatings prepared on different substrates: (**f**) glass slide, (**g**) wood, (**h**) aluminum, and (**i**) zinc; Self-cleaning performance of the pristine glass slide and ZT/PDMS composite coating in different dye solutions: (**j**) MO, (**k**) RhB, and (**l**) MB.

To further investigate the adaptability of the superhydrophobic coating to different substrates and its self-cleaning performance, the coating was deposited on various substrates, including glass slides, wood, aluminum, zinc, and paper. The hydrophobicity of the coated substrates was then compared with that of the pristine substrates [57]. As shown in Figure 6a–e, the superhydrophobic coatings fabricated on different substrate surfaces exhibited continuous, uniform, and complete coverage without obvious cracking or thickness heterogeneity. This indicates that the method possesses good adaptability to various substrates and thus holds great potential for practical applications. On the pristine substrate surfaces, water droplets spread out, demonstrating hydrophilic characteristics. In contrast, the ZT/PDMS composite coating on different substrates exhibited stable superhydrophobic performance, with water droplets maintaining a complete spherical shape on the coating surfaces.

Aluminum ash was used as a model contaminant in simulated tests to evaluate the self-cleaning performance, and the experimental results are presented in Figure 6f–i. On the surfaces of the untreated substrates, the aluminum ash particles remained firmly attached to the substrate surfaces after being wetted by water droplets, and the water flow was ineffective in removing them. This indicated that the unmodified surfaces exhibited strong adhesion to contaminants and lack self-cleaning capability. In contrast, on the ZT/PDMS composite coating surfaces, aluminum ash particles were quickly carried away by rolling water droplets, leaving the coated surfaces clean without observable residual contamination. These results demonstrate that ZT/PDMS composite coatings prepared on different types of substrates exhibit excellent self-cleaning performance and good substrate adaptability.

This superior self-cleaning behavior is primarily attributed to the micro/nano rough structures constructed on the coating surface and the low surface energy imparted by PDMS. The hierarchical structures effectively trap air at the solid–liquid interface, forming a stable air cushion that maintains the water droplets in a typical Cassie–Baxter wetting state. As a result, the actual contact area between the water droplets and the solid surface is significantly reduced, leading to a low sliding angle and low adhesion force [58]. When water droplets roll across the surface, contaminant particles are readily captured and carried away by the droplets, thereby achieving a self-cleaning effect that resembles the “lotus leaf effect” [59].

To further investigate the self-cleaning performance of the ZT/PDMS composite coating, a pristine glass slide and the composite coating were separately immersed in three dye solutions (MO, RhB and MB). After immersion, the samples were taken out and the residual dye liquids on the coating surfaces were observed. As shown in Figure 6j–l, all three dye solutions left obvious liquid residues on the pristine glass slide. The droplets spread and adhered to the glass slide surface, exhibiting typical hydrophilic characteristics. In contrast, the ZT/PDMS composite coating showed almost no residual dye solution on its surface. In summary, the ZT/PDMS composite coating can impart excellent self-cleaning performance to various substrates, effectively avoiding performance degradation caused by contaminant accumulation. This demonstrates its promising application potential.

### 3.4. Photocatalytic Performance and Zn^2+^ Release of ZT/PDMS Composite Coating

**Figure 7:**
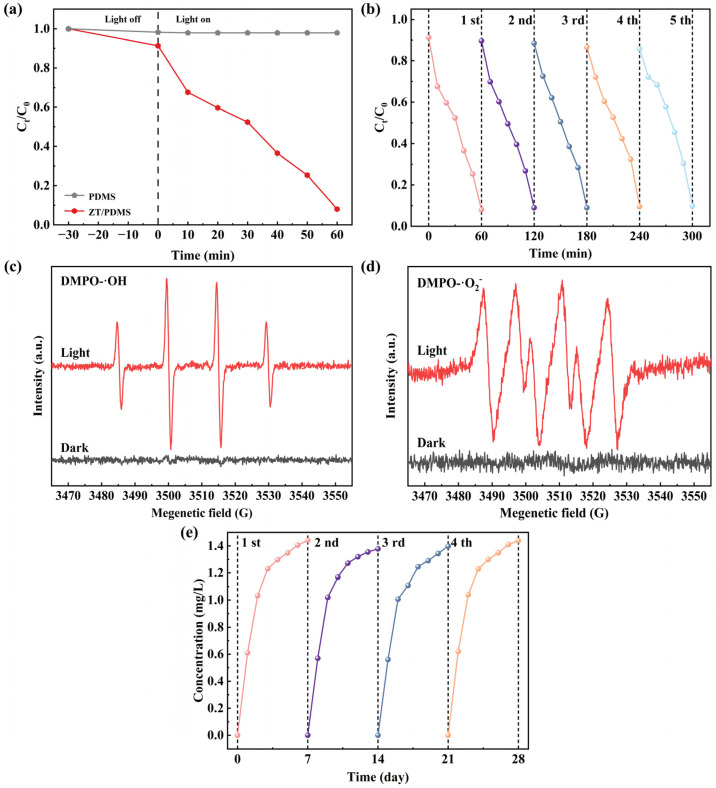
(**a**) Photocatalytic degradation of PDMS coating and ZT/PDMS composite coating; (**b**) Photocatalytic cycling stability of ZT/PDMS composite coating; EPR spectra of (**c**) DMPO-·OH, (**d**) DMPO-·O_2_^−^ under dark and visible light illumination; (**e**) Zinc ion release profiles of ZT/PDMS composite coating.

The photocatalytic degradation performance and cycling stability of the coatings were evaluated using MO as a model pollutant. As shown in Figure 7a, the PDMS coating alone showed no photocatalytic activity. In contrast, the ZT/PDMS composite coating exhibited excellent photocatalytic degradation performance. After 60 min of UV irradiation, the composite coating achieved a degradation efficiency of 91.98% for MO. As shown in Figure 7b, after five consecutive cycles, it still maintained a high degradation efficiency of over 90%, with no significant loss in photocatalytic activity. These results demonstrate that the ZT/PDMS composite coating possesses good photocatalytic degradation performance and good stability over repeated photocatalytic cycles. The excellent photocatalytic performance is attributed to the formation of a Type-II heterojunction between ZnO and TiO_2_. The staggered band alignment enables photoexcited electrons to migrate from the conduction band (CB) of ZnO to the CB of TiO_2_, while photogenerated holes transfer from the valence band (VB) of TiO_2_ to the VB of ZnO under light irradiation. This spatial separation of photogenerated electrons and holes effectively suppresses charge recombination [60].

To detect the active species, EPR spectroscopy was conducted with 5,5-dimethyl-1-pyrroline N-oxide (DMPO) as the spin trap to capture hydroxyl radicals (·OH) and superoxide radicals (·O_2_^−^) [61], and the results are shown in Figure 7c,d. Under dark conditions, no characteristic signals were observed for either DMPO-·OH or DMPO-·O_2_^−^, indicating negligible generation of reactive oxygen species (ROS) in the absence of light. Upon visible light irradiation, the DMPO-·OH spectrum exhibited a typical four-line signal with an intensity ratio of 1:2:2:1, confirming the formation of ·OH. Meanwhile, the DMPO-·O_2_^−^ spectrum showed a characteristic sextet signal, suggesting the generation of ·O_2_^−^ [62]. These results demonstrated that the ZT/PDMS composite coating efficiently produces ROS under visible light illumination. The generation of ·O_2_^−^ and ·OH is attributed to the Type-II ZnO@TiO_2_ heterojunction. Under visible light, photogenerated electrons migrate to TiO_2_ and reduce O_2_ to ·O_2_^−^, while holes migrate to ZnO and oxidize H_2_O/OH^−^ to ·OH. This charge separation minimizes recombination and enables efficient ROS production [63].

The ZT/PDMS coating was immersed in PBS (pH = 7.4, 37 °C) for 7 days, during which the Zn^2+^ concentration in the solution was measured at regular intervals. This immersion–measurement cycle was repeated four times, with the coating being transferred to fresh PBS at the end of each cycle to simulate long-term service conditions. The Zn^2+^ release results are shown in Figure 7e. In all four cycles, a similar trend was observed: the Zn^2+^ concentration increased over time, but the release rate gradually decreased and leveled off after three days. Notably, no significant attenuation in Zn^2+^ concentration occurred with increasing cycle number, indicating the sustained release capability of the coating. Moreover, the Zn^2+^ concentration consistently remained below the maximum permissible level (3 mg/L) in drinking water recommended by the World Health Organization (WHO).

### 3.5. Antibacterial Adhesion and Bactericidal Performance

**Figure 8:**
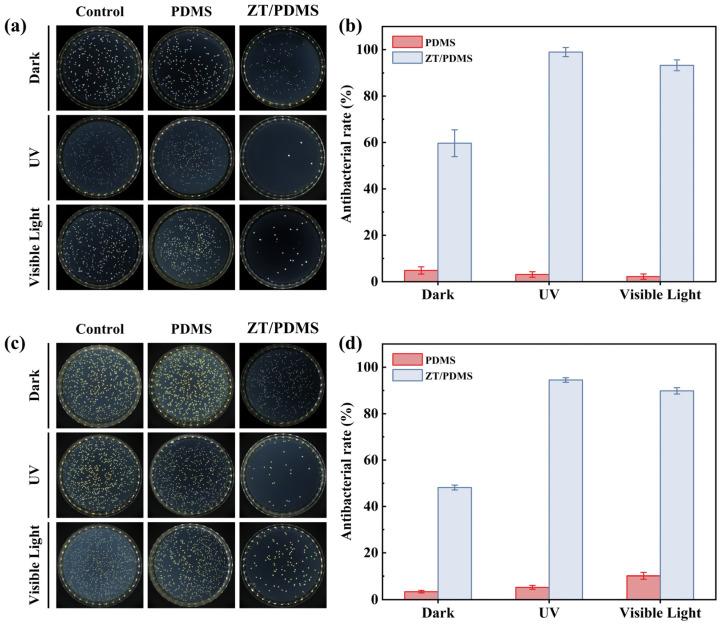
Antibacterial performance of the control, PDMS coating, and ZT/PDMS composite coating under dark, UV light, and visible light conditions. (**a**) Typical plate images and (**b**) corresponding antibacterial rates against *A. baumannii*; (**c**) typical plate images and (**d**) corresponding antibacterial rates against *S. aureus*.

In this study, a pristine glass slide was used as a control to further evaluate the antibacterial performance of the PDMS coating and the ZT/PDMS composite coating against *A. baumannii* and *S. aureus* under dark, UV, and visible light conditions. Figure 8a,b and Figure 8c,d show representative photographic images of the bacterial colonies and the corresponding antibacterial rates for *A. baumannii* and *S. aureus*, respectively. The results indicate that under dark, UV, and visible light conditions, the PDMS coating exhibited no significant antibacterial activity, with a large number of bacterial colonies visible on the agar plates. This was attributed to the absence of any antibacterial functional components in the PDMS coating itself.

In contrast, after treatment with the ZT/PDMS composite coating, the number of bacterial colonies on the plates was markedly reduced, and the antibacterial rate was significantly enhanced. Specifically, under dark conditions, the antibacterial rates of the ZT/PDMS composite coating against *A. baumannii* and *S. aureus* reached 59.7% and 48.14%, respectively, which was primarily attributed to the release of Zn^2+^ ions. Under UV irradiation, the antibacterial rates of the composite coating against *A. baumannii* and *S. aureus* were 98.96% and 94.50%, respectively. Under visible light irradiation, the antibacterial rates of the composite coating against *A. baumannii* and *S. aureus* were 93.22% and 90.83%, respectively. This enhanced performance was ascribed to the ZnO@TiO_2_ heterojunction, which generates a large amount of reactive oxygen species (ROS) upon light excitation, thereby endowing the coating with highly efficient photocatalytic sterilization capability. The synergistic action of ROS production and Zn^2+^ release resulted in a highly efficient antibacterial effect [64].

**Figure 9:**
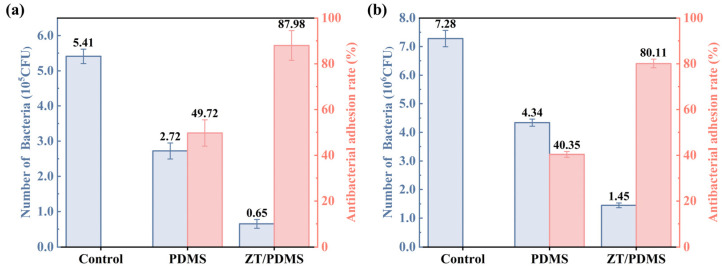
Bacterial adhesion numbers and anti-adhesion rates of different samples against (**a**) *A. baumannii* and (**b**) *S. aureus*.

To assess the antibacterial adhesion performance of the coatings, a pristine glass slide, the PDMS coating, and the ZT/PDMS composite coating were immersed in a bacterial suspension. The number of adherent bacteria on each surface was measured, and the corresponding antibacterial adhesion rates were calculated. The results are shown in Figure 9. The PDMS coating exhibited antibacterial adhesion rates of 49.72% and 40.35% against *A. baumannii* and *S. aureus*, respectively, indicating a moderate antibacterial adhesion ability. However, due to the lack of a micro/nanostructured rough surface, the PDMS coating did not achieve a superhydrophobic state, and consequently, its antibacterial adhesion rate remained relatively low. In contrast, the ZT/PDMS coating achieved antibacterial adhesion rates of 87.98% and 80.11% against *A. baumannii* and *S. aureus*, respectively, exhibiting excellent antibacterial adhesion performance.

This improvement is attributed to the increased surface roughness introduced by the ZT nanoparticles. The combination of micro/nano rough structures and low surface energy imparts superhydrophobicity to the coating. The resulting air layer at the solid–liquid interface significantly reduces the actual contact area between bacteria and the coating surface, thereby further minimizing initial bacterial attachment [13].

### 3.6. Antibacterial Mechanism of the ZT/PDMS Composite Coating

**Figure 10:**
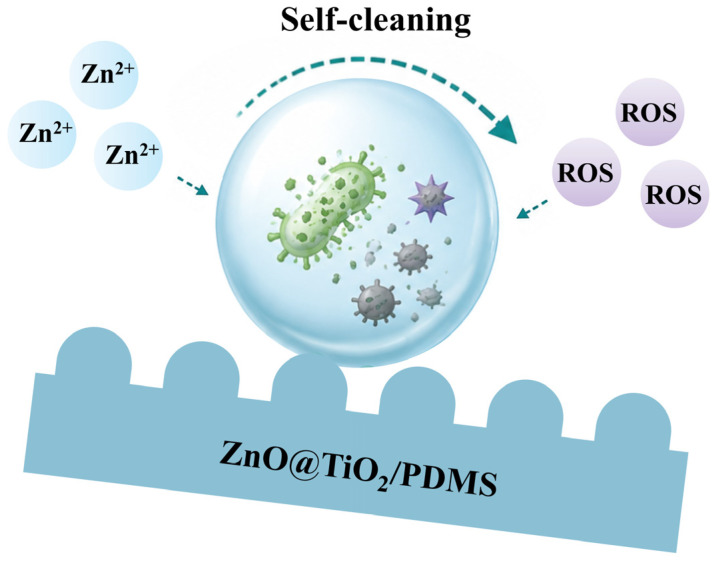
Antibacterial mechanism of ZT/PDMS composite coating: synergistic effects of anti-adhesion, sterilization, and self-cleaning.

The excellent antibacterial performance of the ZT/PDMS composite coating is attributed to the synergistic effect of “anti-adhesion, sterilization, and self-cleaning”, as shown in Figure 10. This mechanism can be summarized in the following three aspects:

(1) Passive anti-adhesion mechanism: The micro/nano rough structures constructed on the coating surface by ZT nanoparticles, combined with the low surface energy of PDMS, endows the coating with exceptional superhydrophobicity. The air layer trapped at the interface between water droplets and the coating surface significantly reduces the actual contact area with bacteria, thereby effectively inhibiting initial bacterial adhesion and biofilm formation.
(2) Active sterilization mechanism: Once bacteria breach the physical barrier created by the superhydrophobic surface, the uniformly dispersed ZT nanoparticles on the coating surface can exert an active killing effect. The Zn^2+^ ions released from the coating are electrostatically attracted to the negatively charged bacterial surfaces. These ions, together with the ROS generated by photocatalysis, disrupt the cell membrane potential and penetrate the cell membrane, leading to extensive leakage of intracellular contents. Once inside the cells, Zn^2+^ and ROS damage bacterial proteins and DNA, induce oxidative stress, and interfere with normal bacterial metabolism, thereby achieving effective killing of attached bacteria.
(3) Self-cleaning mechanism: The outstanding superhydrophobicity of the coating also confers self-cleaning properties. When water droplets roll across the surface, they readily remove dead bacteria and environmental contaminants, effectively preventing the accumulation of dead bacteria that could otherwise serve as attachment sites for subsequent live bacterial colonization. This mechanism not only maintains the surface cleanliness of the coating but also ensures the sustained effectiveness of its anti-adhesion and active bactericidal functions over long-term use.

## 4. Conclusions

In this study, ZnO@TiO_2_ nanoparticles were prepared via a sol–gel method, and a ZT/PDMS composite coating was fabricated with PDMS serving as both a binder and a hydrophobic modifier. The preparation method exhibited good adaptability to various substrates and endowed the coating with excellent mechanical durability, chemical stability, and self-cleaning performance. Owing to the synergistic mechanism of “anti-adhesion, active sterilization, and self-cleaning”, the coating demonstrated outstanding antibacterial adhesion and antibacterial activity, achieving an antibacterial adhesion rate of 87.98% and 80.11% against *A. baumannii* and *S. aureus*, respectively. Moreover, under both UV and visible light irradiation, the bactericidal efficiency exceeded 90%. The ZT/PDMS composite coating integrates superhydrophobicity, durability, self-cleaning, and high-efficiency antibacterial properties, providing a promising strategy for the development of long-lasting antibacterial surfaces.

## Data Availability

The original contributions presented in this study are included in the article/Supplementary Material. Further inquiries can be directed to the corresponding authors.

## Supplementary Materials

The following supporting information can be downloaded at: https://www.mdpi.com/article/10.3390/ma19112380/s1, Figure S1: (a) TEM images of ZnO/TiO_2_ nanoparticles; (b) XRD patterns of ZnO and ZnO/TiO_2_ nanoparticles; (c) FTIR spectra of ZnO and ZnO/TiO_2_ nanoparticles. Figure S2: Contact angles of the ZT/PDMS composite coatings prepared with different m (ZT:PDMS). Table S1: Atomic concentration fractions (%) of the ZT/PDMS composite coating surface obtained from XPS analysis. Figure S3: Images of the ZT/PDMS composite coating after cross-cut adhesion test.
