# Supplementary material for: ZnO@TiO2/PDMS Superhydrophobic Antibacterial Coating with Photocatalytic Activity, Durability, and Self-Cleaning Properties
Source: Materials (Basel). 2026 Jun 3;19(11):2380. doi: 10.3390/ma19112380 (PMC13258453; doi:10.3390/ma19112380)
Supplement: Supplementary file 1 [file materials-19-02380-s001.zip › materials-4339356-supplementary.pdf]

materials

$ZnO@TiO_2$/PDMS Superhydrophobic Antibacterial Coating with Photocatalytic Activity, Durability, and Self-Cleaning Properties

Shuyu Yuan [1], Yuan Feng [1], Shuaichao Liang [1], Huidong Cai [2,*] and Qingge Feng [1,*]

1 Guangxi Universities Key Laboratory of Environmental Protection, School of Resources, Environment and Materials, Guangxi University, Nanning 530004, China; yuanshuyu_1@163.com (S.Y.); fengy_cup@163.com (Y.F.); m17877988861@163.com (S.L.)
2 School of Environmental Science and Engineering, Guangdong University of Petrochemical Technology, Maoming 525000, China
* Correspondence: caihuidong1992@163.com (H.C.); fengqg@gxu.edu.cn (Q.F.)

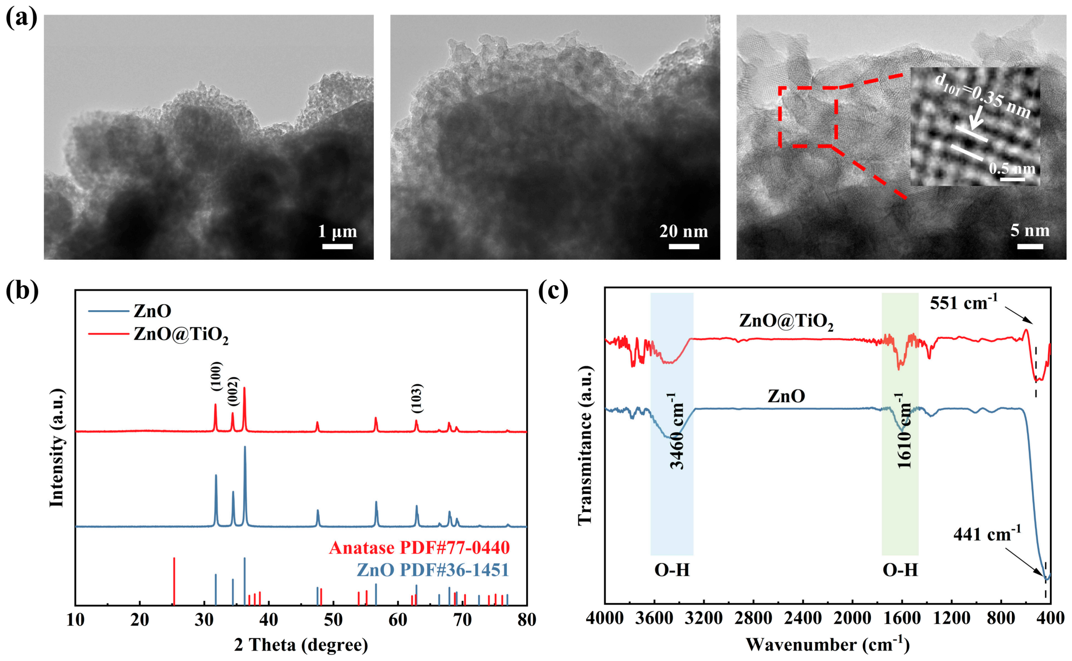

Figure S1. (a) TEM images of ZnO/TiO_2 nanoparticles; (b) XRD patterns of ZnO and ZnO/TiO_2 nanoparticles; (c) FTIR spectra of ZnO and ZnO/TiO_2 nanoparticles.

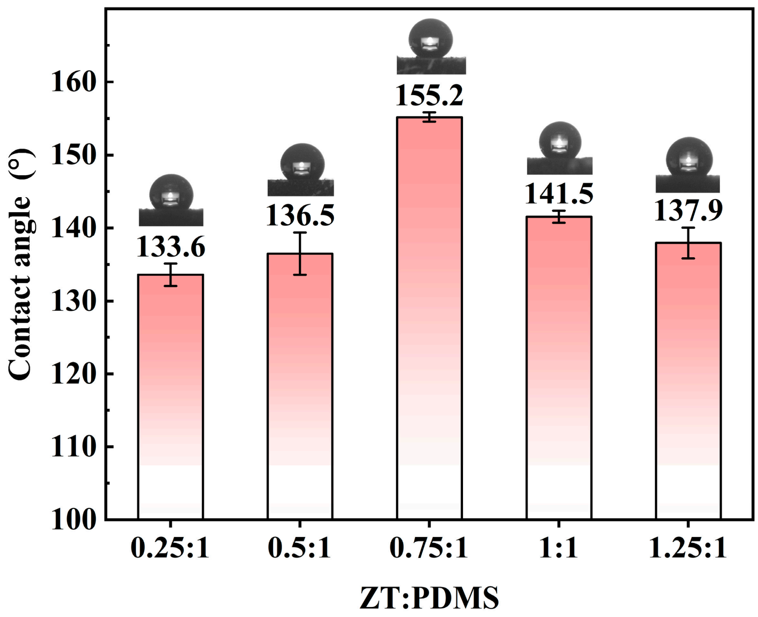

Figure S2. Contact angles of the ZT/PDMS composite coatings prepared with different m (ZT:PDMS).

Table S1. Atomic concentration fractions (%) of the ZT/PDMS composite coating surface obtained from XPS analysis.

Element	Atomic %
O	26.49
C	50.21
Si	21.99
Ti	0.68
Zn	0.63

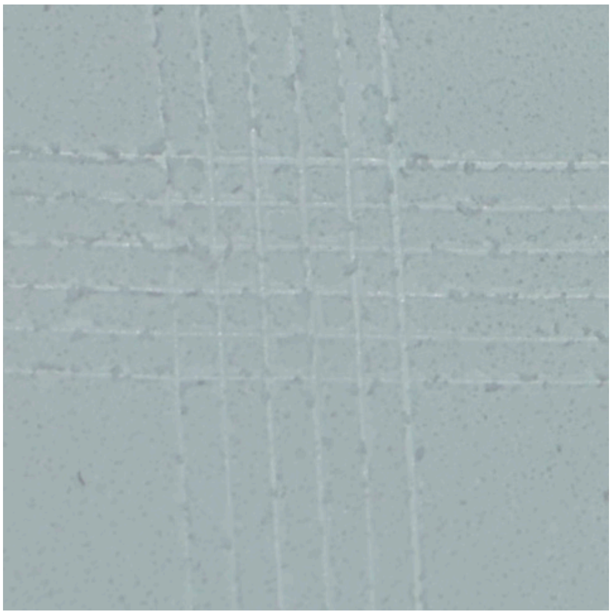

Figure S3. Images of the ZT/PDMS composite coating after cross-cut adhesion test.
